# Are Older Adults Sexually Well: A Comparative Qualitative Study in Portugal and Romania

**DOI:** 10.1093/geroni/igaa057.992

**Published:** 2020-12-16

**Authors:** Sofia von Humboldt, Georgeta Niculescu, Gail Low, Isabel Leal

**Affiliations:** 1 Instituto Superior de Psicologia Aplicada (ISPA), Lisbon, Portugal; 2 Romanian Association for Person Centred Psychotherapy (ARPCP), Bucharest, Romania; 3 Faculty of Nursing, University of Alberta, AB, Edmonton, Canada, Alberta, Canada

## Abstract

Objective: This study aims the perspectives of older adults on their sexual well-being. For this purpose, a qualitative research was carried out, which analyzes older adults’ perspectives on indicators of sexual well-being in Portugal and Romania. Methods: Forty seven older participants aged 65 to 91 years, were interviewed. Participants lived in the community. All the interviews went through content analysis. Results: Preliminary results of content analysis generated 5 themes for the Romanian sample: Supportive relationship (k = .92, p < .01); positive financial situation (k = .91, p < .01); good health (k = 94, p < .01); education (k = .88, p < .01); and family support (k = .89, p < .01); and five themes for the Portuguese sample: Supportive relationship (k = .91, p < .01); demonstration of love (k = .91, p < .01); sharing joint activities (k = 92, p < .01); positive attitude and good humor (k = .91, p < .01); and open communication (k = .99, p < .01); Conclusions: This study highlighted the perspectives of Portuguese and Romanian older adults concerning sexual well-being. For both samples, showing a supportive relationship with a partner was the more frequent theme. Keywords: Content analysis; cross-national; older adults; qualitative study; sexual well-being.

